# First known case of human bertiellosis in a child in Paraná, Brazil

**DOI:** 10.1590/1984-0462/2024/42/2023077

**Published:** 2023-12-18

**Authors:** Nathália Mitsue Kishi, Amanda Parteka de Godoy, Bárbara Luiza Viana Afonso, Cristina Alvarez Mattar, Gabriela Riter Martins de Matos, Lucas Müller Prado, Maria Augusta Kormann, Raphael Ferreira Barbosa, Rodolfo Corrêa de Barros, Andrea Maciel de Oliveira Rossoni

**Affiliations:** a Universidade Federal do Paraná, Curitiba, PR, Brazil.

**Keywords:** Cestode infection, Bertielliasis, Parasitic diseases, Intestinal diseases, parasitic, Infecções por cestoides, Bertielliasis, Doenças parasitárias, Enteropatias parasitárias

## Abstract

**Objective:**

To describe the first known case of human Bertiellosis in Paraná (Brazil).

**Case description:**

A 6-year-old male residing in the Brazilian state of Paraná was suffering from intermittent nonspecific abdominal pain and abdominal distension, associated with expulsion of live tapeworms in his feces for 7 months. He had a history of interaction with monkeys on an island. His first feces analysis was inconclusive, with an initial hypothesis of an atypical *Taenia*. However, after additional research, the parasitologist identified pregnant proglottids of *Bertiella* sp. The patient was initially treated with an unknown dosage of albendazole and nitazoxanide, as it was believed he had been infected with *Taenia* sp. Since the symptoms persisted, praziquantel 10 mg/kg was prescribed without further proglottids elimination.

**Comments:**

Human Bertiellosis is a rare zoonosis, with less than one hundred cases reported. However, it is a cause of chronic abdominal pain and should be kept as a differential diagnosis, especially in cases reporting recurrent tapeworm expulsion in feces and refractory treatment with albendazole.

## RESUMO

**Objetivo:**

Descrever o primeiro caso conhecido de bertielose humana no Paraná, Brasil.

**Descrição do caso:**

Criança de seis anos do sexo masculino, residente no Paraná, Brasil, apresentava dor abdominal inespecífica intermitente e distensão abdominal, associadas à expulsão de helmintos vivos em suas fezes havia sete meses. Tinha um histórico de interação com macacos em uma ilha. Sua primeira análise de fezes foi inconclusiva, com hipótese inicial de uma *Taenia* atípica. No entanto, após pesquisas adicionais, o parasitologista identificou proglótides gravídicas de *Bertiella* sp. O paciente foi inicialmente tratado com uma dosagem desconhecida de albendazol e nitazoxanida, pois se acreditava que havia sido infectado por *Taenia* sp. Diante da persistência dos sintomas, foi prescrito praziquantel 10 mg/kg, sem mais eliminação de proglótides.

**Comentários:**

A bertielose humana é uma zoonose rara, com menos de cem casos relatados. No entanto, é uma causa de dor abdominal crônica e deve ser mantida como diagnóstico diferencial, principalmente nos casos que relatam expulsão recorrente de helmintos nas fezes e que são refratários ao tratamento com albendazol.

## INTRODUCTION

Human bertiellosis is a rare zoonosis with less than a hundred case reports worldwide,^1^ being six of them in Brazil, in the states of São Paulo, Minas Gerais, Goiás, and Pará.^1–6^ Therefore, this is the first case report known of human bertiellosis in the state of Paraná, Brazil.

This infection may be caused by three species of *Bertiella: Bertiella struderi, Bertiella mucronate*, and *Bertiella satyri*.^1,7,8^ Humans are accidental hosts that may acquire this zoonosis by close contact with marsupials, rodents, and mainly non-human primates, such as monkeys. They also may be infected by the accidental ingestion of fruits or soil infected with intermediate hosts of *Bertiella*.^1,9,10^

Bertiellosis is more common in children, since they are more likely to have geophagia and usually have lower hygiene skills.^1,7,10^ These mites can be found in the soil and in the fur of monkeys, dogs, and other animals.^9^ With the increase of deforestation and urbanization, it is getting more common for people to have contact with primates and spaces inhabited by them, which may have caused higher rates of bertiellosis cases.^3,7,11^

It may be asymptomatic or manifest clinical signs such as diarrhea or constipation, abdominal pain, anorexia, weight loss, vomiting and the presence of tapeworms in feces.^9,10^ There are no reports of serious complications, however, chronic pain can lead to pain hypersensitivity, anxiety, stress, attention deficit disorders,^12^ in addition to iatrogenesis by over-investigation, misdiagnosis, and inappropriate treatment.^13^ Diagnosis is made by combining epidemiology, symptoms, and a morphometric analysis of the remaining structures of the parasite, identifying the pregnant proglottid and the morphology of *Bertiella*’s egg.^9^ Treatment can be done with praziquantel^1,10^ or niclosamide,^9^ however, there are reports of resistance to the latter.^1,14^

This is a report of a child with human bertiellosis whose treatment was delayed due to a difficult diagnosis.

## CASE REPORT

Male, 6 years old, resident in a rural area of Paraná (Brazil). His mother reported that 7 months earlier the patient started with an intermittent nonspecific abdominal pain, combined with abdominal distension and live tapeworms in his feces. First, he was evaluated by local clinicians, who treated the case as a common infection by *Taenia solium* with albendazole and nitazoxanide (unknown dosage). Since he persisted with the same symptoms, he was prescribed praziquantel 10 mg/kg in a single dose and was referred to our service, as it is a tertiary teaching hospital.

At the consultation, the child was eutrophic (high 119 cm; weight 24 kg) and both general and specific physical examinations were normal. Laboratory testing demonstrated hemoglobin 12 g/dl, white cells 10.700 WBC/dl (eosinophil 2% – 214), erythrocyte sedimentation rate of 3 mm, and negative C-reactive protein.

**Figure 1:**
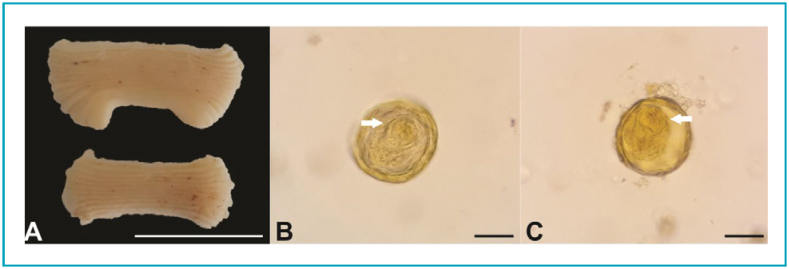
A. Macroscopic view of proglottids of *Bertiella* sp. obtained from the stools of the patient. B-C. Eggs found in the pregnant proglottids with typical pyriform apparatus (arrows). Scale bars: A=1 cm, B-C=20 μm.

Initial analysis of the feces sample was inconclusive (with a sample that the mother had from before the treatment) and required further investigation of possible pathogens. The first hypothesis raised by the microbiologist was an atypical *Taenia*. However, after additional research in the same sample, the microbiologist identified pregnant proglottids of *Bertiella sp* (Figure 1).

Since de use of praziquantel, he had no more complaints and no more proglottids were expelled in the feces. When actively questioned about contact with primates, the only interaction with monkeys was on an island, while the child was fishing with his parents, and he had indirect contact with the animals. No one else in the family had similar symptoms.

Upon return, two months after treatment, the patient was asymptomatic, with a negative parasitological stool test. The mother signed a written consent form authorizing the publication of the case and the report was approved by the Ethics Committee of the Institution (CAAE: 67967123.8.0000.0096).

## DISCUSSION

Human Bertiellosis is an infection caused by the accidental ingestion of Oribatid mites contaminated with cysticercoid larvae of a helminth of the *Anoplocephalidae* family, genus *Bertiella*. Three species from this genus are capable of infecting humans: *Bertiella studeri, Bertiella mucronate*, and *Bertiella satyri*, recently described as different species.^1,7,8^

These species have non-human primates as their main definitive hosts and most human cases have a history of contact with these animals, as seen in this patient.^1,2,9,10^ With urbanization and deforestation, there has been an expansion of the areas of interaction between humans and these animals, accompanied by an increase of human and non-human primates infections.^3,7,11^ With this in mind, it is essential to inquire about travels and contact with primates when investigating the patient’s history.^1^

The parasite cycle occurs as follows: adult tapeworms live in the small intestine of the definitive host, non-human primates. These definitive hosts liberate eggs or pregnant proglottids in the environment through their feces. These *Bertiella* eggs are consumed by Oribatid mites, the intermediate hosts that live in the soil and fruits. The cycle is completed with the consumption of these contaminated soil and fruits by primate definitive hosts.^1^

This disease is an uncommon parasitic disease, with less than one hundred reports worldwide,^1^ of which six were identified in Brazil, in the states of São Paulo, Minas Gerais, Goiás, and Pará.^1–6^ This report, therefore, is the first known case of *Bertiella* spp. infection in Paraná, a state located in southern Brazil.

The infection is more common in children, owing to the habit of geophagia.^1,7,10^ Human bertiellosis can be asymptomatic^1,9^ or present with chronic abdominal pain, diarrhea, anorexia, weight loss,^7,9,10,14^ abdominal distention,^1,6,10^ constipation,^9,10,14^ vomiting,^3,5,10,14^ dyspepsia^1,3,5^, and perianal pruritus.^7,9,10^ However, these are nonspecific symptoms and can be found in other diseases.^7^ There may also be worms in the stool,^7,9,10^ which often leads to seeking medical attention.^1^

Diagnosis is made by combining epidemiology, symptoms and a morphometric analysis of the remaining structures of the parasite.^9^ The scolex of *Bertiella* spp. carry four large cup-shaped suckers and no rostellum or hooks are present. The proglottids (Figure 1A) are much wider than they are long (6–15 mm×1–3 mm) and show a lateral genital pore. The eggs (Figures 1B and 1C) are spherical (35–62 μm) with a filamentous vitelline membrane and a well-developed pyriform apparatus surrounding oncospheres.^1^

It is possible that this disease is under-reported, since the diagnosis is made through the morphological evaluation of the patient’s feces and some cases can be mistakenly identified as another zoonosis due to the apparent similarity with other tapeworms.^10^ Furthermore, this is a rare parasitic disease, with only six reports in Brazil, in other words, there is still scarce knowledge about it. Moreover, patients with human Bertiellosis are mainly children,^1,7,10^ which can interfere with the collection of information about the clinical history and make the diagnosis of Bertiellosis difficult.

The difficulty in its isolation can contribute to the chronicity of the disease.^10^ There are no reports of serious complications, however, chronic pain can lead to pain hypersensitivity, anxiety, stress, attention deficit disorders,^12^ in addition to iatrogenesis by over-investigation, misdiagnosis, and inappropriate treatment.^13^

Treatment is conventionally carried out with praziquantel.^1,10^ Some reports described in the literature demonstrate that a single dose of praziquantel 10 mg/kg^5,6,9^ is capable of successfully eliminating the parasite. Another drug also used is niclosamide (1–2 g),^9^ however, there are reports of treatment failure with this drug.^1,14^ Furthermore, there have been cases in which the use of albendazole was not effective in treating bertiellosis,^5,10^ like this report, but there have been no reports of patients refractory to praziquantel to this date.^1,5,6,14^

Due to the lack of awareness regarding *Bertiella* spp. there is a possibility that cases of this zoonosis are underreported, causing a delay in treatment. Considering this, Bertiellosis should be considered as a possible diagnosis in cases suggestive of tapeworm infection that are refractory to albendazole. For this diagnosis to be made, it is important to ask animal contact history and to perform a morphometric analysis of the remaining structures of the parasite. This elucidates the importance of spreading awareness about this disease to both physicians and microbiologists.
